# Prediabetes Is Associated With Brain Hypometabolism and Cognitive Decline in a Sex-Dependent Manner: A Longitudinal Study of Nondemented Older Adults

**DOI:** 10.3389/fneur.2021.551975

**Published:** 2021-02-19

**Authors:** Erin E. Sundermann, Kelsey R. Thomas, Katherine J. Bangen, Alexandra J. Weigand, Joel S. Eppig, Emily C. Edmonds, Christina G. Wong, Mark W. Bondi, Lisa Delano-Wood

**Affiliations:** ^1^Department of Psychiatry, University of California, San Diego, La Jolla, CA, United States; ^2^Veterans Affairs San Diego Healthcare System, San Diego, CA, United States; ^3^San Diego State University/University of California, San Diego (SDSU/UCSD) Joint Doctoral Program in Clinical Psychology, San Diego, CA, United States

**Keywords:** prediabetes, sex, brain metabolism, Alzheimer's disease, hippocampal volume, amyloid-beta, phosphorylated tau, cognitive function

## Abstract

Although type 2 diabetes is a well-known risk factor for Alzheimer's disease (AD), little is known about how its precursor—prediabetes—impacts neuropsychological function and brain health. Thus, we examined the relationship between prediabetes and AD-related biological and cognitive/clinical markers in a well-characterized sample drawn from the Alzheimer's Disease Neuroimaging Initiative. Additionally, because women show higher rates of AD and generally more atherogenic lipid profiles than men, particularly in the context of diabetes, we examined whether sex moderates any observed associations. The total sample of 911 nondemented and non-diabetic participants [normal control = 540; mild cognitive impairment (MCI) = 371] included 391 prediabetic (fasting blood glucose: 100–125 mg/dL) and 520 normoglycemic individuals (age range: 55–91). Linear mixed effects models, adjusted for demographics and vascular and AD risk factors, examined the independent and interactive effects of prediabetes and sex on 2–6 year trajectories of FDG-PET measured cerebral metabolic glucose rate (CMRglu), hippocampal/intracranial volume ratio (HV/IV), cerebrospinal fluid phosphorylated tau-_181_/amyloid-β_1−42_ ratio (p-tau_181_/Aβ_1−42_), cognitive function (executive function, language, and episodic memory) and the development of dementia. Analyses were repeated in the MCI subsample. In the total sample, prediabetic status had an adverse effect on CMRglu across time regardless of sex, whereas prediabetes had an adverse effect on executive function across time in women only. Within the MCI subsample, prediabetic status was associated with lower CMRglu and poorer executive function and language performance across time within women, whereas these associations were not seen within men. In the total sample and MCI subsample, prediabetes did not relate to HV/IV, p-tau_181_/Aβ_1−42_, memory function or dementia risk regardless of sex; however, among incident dementia cases, prediabetic status related to earlier age of dementia onset in women but not in men. Results suggest that prediabetes may affect cognition through altered brain metabolism, and that women may be more vulnerable to the negative effects of glucose intolerance.

## Introduction

Type 2 diabetes (T2D) is a well-established risk factor for accelerated cognitive decline, mild cognitive impairment (MCI) and Alzheimer's disease (AD) (1–6). Additionally, there are consistent reports of associations between T2D and AD-related brain changes in non-demented older adults (7–18). For example, T2D is associated with smaller brain volumes, accelerated rates of brain atrophy, reduced cerebral blood flow in predilection sites for AD pathology (e.g., medial temporal lobe, inferior parietal regions), reductions in cerebral glucose metabolic rate (CMRglu) (7–15, 19), and higher cerebrospinal fluid (CSF) levels of the AD pathological marker hyperphosphorylated tau (p-tau) (16–18). Even among nondiabetics, elevated blood glucose has been associated with more severe AD pathology (i.e., higher medial temporal lobe neurofibrillary tangle pathology) (20). Associations between T2D and the other AD pathological hallmark characteristic, amyloid-β (Aβ), are less clear (8, 17, 21, 22).

Despite these well-established associations with T2D, far less is known about the effects of its precursor condition, prediabetes, on brain health and cognitive function. The impact of prediabetes on the brain is of great public health significance considering that half of adults aged 60 and older are estimated to have prediabetes (23), yet most are unaware that they have this condition. Although enough insulin is produced to thwart a T2D diagnosis, prediabetes is characterized by exposure to abnormally high levels of insulin for years, which results in insulin resistance and, in turn, impaired fasting glucose (24, 25). Because insulin and insulin-like growth factors regulate many biological processes such as axonal growth, protein synthesis, and gene expression (26, 27), insulin resistance and the resultant oxidative stress adversely affect all of these processes, which often occur before onset of overt T2D (28–30). In fact, older adults (age ≥ 65) with prediabetes have shown a two times greater risk of incident AD over 3,691 person-years (24); however, the specific effect of prediabetes on cognitive function and brain health in older adults without dementia is unclear. In a small study of participants with prediabetes (*n* = 23), there was an association between greater insulin resistance and a reduction in CMRglu in frontal, parietotemporal, and cingulate regions (14). A recent meta-analysis demonstrated that CSF levels of Aβ, p-tau and total tau (t-tau) were not associated with prediabetes status overall; however, prediabetes was associated with lower Aβ levels (indicative of greater Aβ plaque deposition in the brain) and higher t-tau levels among participants with memory impairment, suggesting that prediabetes may accelerate AD progression (18). Larger, longitudinal studies, such as the current study, are needed to understand the links between prediabetes, AD brain changes, and cognitive decline that may contribute to AD risk.

There are important, well-documented sex differences in both AD and in the manifestation of T2D. Compared to men, women have a higher prevalence of AD (31–33) and show a two times faster rate of decline in MCI (34) and greater severity in clinical AD dementia (35–37). In terms of T2D, women typically have a more favorable cardiovascular risk profile than men but lose that advantage with menopause (38–40) and/or T2D onset (41). In fact, the risk of cardiovascular events among individuals with T2D is estimated to be 50% greater in women than in men (42, 43), although this estimate varies across studies (44, 45). In a study of sex differences in risk factors for myocardial infarction (*N* = 15,152 cases and 14,820 controls), diabetic women were at almost double the risk of myocardial infarction compared to diabetic men (43). The more adverse cardiovascular profile in diabetic women vs. diabetic men is thought to be due to women showing greater lipid imbalances and more severe insulin sensitivity and inflammation compared to men (42, 44, 46). These differences in women vs. men present many years before T2D diagnosis, suggesting that these differences exist at the prediabetes stage (42, 44, 46).

Despite these sex differences in AD and in the T2D/prediabetes profile, studies have not yet examined sex differences in the relationship between these conditions and AD outcomes. Herein, we used longitudinal data drawn from the Alzheimer's Disease Neuroimaging Initiative (ADNI) to examine the relationship between prediabetes and changes in AD biological (hippocampal volume, brain glucose metabolism, AD CSF biomarkers) and cognitive/clinical markers (memory, executive function, and language performance, progression to dementia) in nondemented, middle-aged and older adults, and whether these relationships are moderated by sex. This research will help provide insight into (1) the impact of prediabetes on cognitive function in older adults without dementia, (2) the neural mechanisms underlying the link between prediabetes/T2D and AD, and (3) whether these associations differ in men vs. women. We hypothesized that prediabetic status will be associated with more advanced AD biomarkers and faster cognitive decline over time, particularly with frontal-mediated cognition (e.g., executive function) that is commonly influenced by vascular mechanisms (47–50) compared to normoglycemic individuals, and that these associations would be stronger in women vs. men.

## Materials and Methods

### Participants and Data Source

Data were extracted from the ADNI database. ADNI data is publically available at adni.loni.usc.edu. ADNI is a longitudinal, multi-site, cohort study that began in 2003 as a public-private partnership. Information about ADNI can be found at www.adni-info.org. The primary goal of ADNI has been to test whether serial neuroimaging measures and other biological and clinical markers can be combined to measure the progression of MCI and early AD. ADNI study visits involve neuroimaging, neuropsychological and clinical assessments. This research was approved by the Institutional Review Boards of all participating sites, and written informed consent was obtained for all participants. The general enrollment inclusion/exclusion criteria for ADNI have been described elsewhere (51). This specific study included participants who had the following data points at their baseline ADNI visit: (1) fasting glucose data as part of the FDG-PET imaging, (2) at-least one of the AD markers of interest and (3) relevant covariate data (*n* = 1,294). We excluded participants with a dementia diagnosis at baseline (*n* = 240). Given the limited number of participants with diabetes in ADNI and our interest in the relationship between prediabetes and AD, we also excluded those who were diabetic according to self-reported medical history and criteria from the World Health Organization (WHO), including fasting blood glucose levels ≥ 126 mg/dL and/or self-reported diabetic medication (*n* = 145) (52). Sample size varied by AD marker with the largest sample size including 911 participants who had brain glucose metabolism data since measuring fasting blood glucose was conducted as part of the [18F]fludeoxyglucose (FDG)-PET scan. See Table 1 for sample size for each AD marker by sex and prediabetes status. Among the largest sample (*N* = 911), 46% were female and 41% were MCI with 391 prediabetic and 520 normoglycemic individuals (144 participants from ADNI1 and 765 from ADNI2/GO).

**Table 1:** Baseline sample size for each AD marker in the overall and MCI subsample as a function of sex and prediabetes status.

**Diagnostic Group/AD marker**	**Total Sample, *N***	**Maximum follow-up period examined (months)**	**Women**		**Men**	
			**Prediabetic, *N***	**Normo-glycemic, *N***	**Prediabetic, *N***	**Normo-glycemic, *N***
**Overall**						
HV/IV	803	36	154	222	194	233
CMRglu	911	72	167	252	222	270
p-tau_181_/Aβ_1−42_ ratio	718	48	134	206	172	206
AVLT Z-scores	910	60	167	251	222	270
TMT Part B Z-scores	902	60	165	249	219	269
BNT Z-scores	910	60	167	251	222	270
Incident dementia	850	All available	154	239	203	254
analysis^*^						
**MCI**						
HV/IV	330	24	50	89	98	93
CMRglu	354	36	58	104	81	111
p-tau_181_/Aβ_1−42_ ratio	290	48	46	88	76	80
AVLT Z-scores	370	48	58	103	98	111
TMT Par B Z-scores	368	48	58	103	96	111
BNT Z-scores	370	48	58	103	98	111
Incident dementia	341	all available	55	96	87	103
analysis^*^						

* *Cox proportional hazards models estimating incident dementia rates were conducted in participants with at-least one follow-up visit. MCI, Mild Cognitive Impairment; HV/IV, hippocampal/intracranial volume x 10^3^; CMRGlu, cerebral metabolic glucose rate; p-tau_181_/Aβ_1-42_, phosphorylated tau/amyloid-β; AVLT, Rey Auditory Verbal Learning Test; TMT, Trail Making Test; BNT, Boston Naming Test*.

### Clinical Diagnosis

Diagnosis of NC vs. MCI was based on the Jak/Bondi diagnostic method (53). This method included six neuropsychological tests representing three cognitive domains: Trail-Making Tests A and B (psychomotor speed/executive function), Category Fluency and Boston Naming Test (language) and the Rey Auditory Verbal Learning Test (AVLT) Delayed Recall and Recognition Tests (episodic memory). An impaired score was defined as >1 SD below the age-corrected normative mean. MCI diagnosis required one of three criteria: (1) impaired score on two tests within a cognitive domain; (2) one impaired score in each of the three cognitive domains; and/or (3) a score of 9 on the Functional Assessment Questionnaire indicating dependence in at-least three daily activities. If no criterion was met, participants were considered cognitively normal. Diagnostic criteria for dementia was based on the standard NINCDS/ADRDA criteria (54).

### Prediabetes Classification

As part of the FDG-PET protocol, participants had blood drawn following a fast of at-least 4 h (water only) and blood glucose was measured. Prediabetic status was ascribed based on blood draw from the baseline visit and was defined as fasting glucose blood levels of 100–125 mg/dL based on guidelines from the American Diabetes Association (55).

### AD Markers

#### Structural MRI

Structural MRI scans were collected on a 1.5T scanner according to a standardized protocol (56). Hippocampal volume data were analyzed using FreeSurfer version 4.3 (https://surfer.nmr.mgh.harvard.edu) at the University of California–San Francisco (57). To control for individual differences in head size, we created a ratio measure of hippocampal volume to intracranial volume (HV/IV) using the formula, hippocampal volume/intracranial volume × 10^3^.

#### Cerebral Metabolic Glucose Rate

CMRglu was measured by FDG-PET. Images were preprocessed following a standard procedure described at http://adni.loni.usc.edu/methods/pet-analysis/pre-processing/. ADNI investigators at the University of California–Berkeley established a “Meta-Regions of Interest” (MetaROI) of brain regions that commonly demonstrate metabolic changes in MCI/AD and correlate with cognitive performance in a meta-analysis (58, 59). The “MetaROI” was comprised of bilateral posterior cingulate gyrus, bilateral angular gyrus, and middle/inferior temporal gyrus. Standardized uptake value ratios (SUVRs) were calculated by averaging FDG uptake across the MetaROI and dividing by a reference region of pons and cerebellum (58, 59). The protocol for image analysis is described in http://adni.loni.usc.edu/methods/pet-analysis-method/pet-analysis/#pet-pre-processing-container.

#### Cerebrospinal Fluid Phosphorylated-tau-181 (p-tau_181_)/Aβ_1−42_ Ratio

We examined the ratio of CSF levels of hyperphosphorylated tau-181 (p-tau_181_) to Aβ_1−42_ proteins (p-tau_181_/Aβ_1−42_ ratio) which has been shown to predict cognitive decline in individuals with MCI (60).

#### Cognitive Performance

Within the ADNI neuropsychological test battery, we examined tests measuring cognitive domains that typically show impairments early in the AD trajectory including language [Boston Naming Test (BNT)], executive function [Trail Making Test (TMT) Part B], and episodic memory (AVLT - Delayed Recall) (61). Individual scores from these tests were converted to z-scores based on age- and education-adjusted regression coefficients derived from a normative control group (*n* = 328) that remained cognitively normal (as identified via ADNI criteria) throughout follow-up in ADNI. The TMT Part B z-scores were multiplied by −1 so that higher scores indicated better performance.

### Statistical Analyses

Differences in sample characteristics and baseline AD markers between sex and prediabetes status were examined using independent *t*-tests for continuous variables and Chi-square tests for categorical variables. We conducted linear mixed effects models with a random intercept to examine the separate and interactive effects of sex and baseline prediabetes status on (1) AD biological (HV/IV, CMRglu, p-tau_181_/Aβ_1−42_ ratio) and clinical markers (AVLT, BNT and TMT Part B z-scores) across time and (2) the change in these markers over 2–6 years (depending on the follow-up period that varied by AD marker in ADNI; see Table 1). The effect of the predictors on change in AD markers was modeled by examining all two- and three-way interactions with time, which was modeled continuously (i.e., sex X time, prediabetes X time and sex X prediabetes X time). Models with three-way interactions (i.e., sex by prediabetes by time) controlled for all two-way interactions and main effects. Separate linear mixed effects models were conducted for each AD-marker outcome and each model included data from time points in ADNI that had at-least 10 participants per group (i.e., female/male diabetic/normoglycemic) resulting in at-least 40 participants total at a time point. Among participants with at-least one follow-up visit (*N* = 850), Cox proportional hazards models estimated hazard ratios (HR) and 95% confidence intervals (CI) for risk of developing dementia during follow-up as a function of sex, prediabetes, and sex X prediabetes. Among those who developed dementia during follow-up, we conducted a linear regression to examine the effect of sex, prediabetes and sex X prediabetes on the age at dementia-onset. In all analyses, male sex was compared to female sex (reference group) and the prediabetes group was compared to the normoglycemic group (reference group). Significant interactions were probed via sex- and prediabetes-stratified analyses. Interaction terms were removed from the model if *p* ≥ 0.10 in order to assess main effects. Covariates in statistical models included demographic variables (age, education, race/ethnicity), apolipoprotein ε4 allele (APOE4), and indices of cardiovascular risk given sex differences in cardiovascular risk profiles beyond prediabetes/T2D (38–40). We selected cardiovascular risk indices as covariates that commonly cluster with diabetes (62) and are available in ADNI including body mass index (BMI), pulse pressure (systolic blood pressure – diastolic blood pressure), and history of any self-reported cardiovascular event (e.g., hypertension, hyperlipidemia, atrial fibrillation, coronary artery disease, and stroke). In analyses examining risk of dementia and age at dementia-onset, we additionally adjusted for baseline AD biomarkers. Any covariate that was not significant in the multivariable regression model at a *p* ≤ 0.10 threshold level was removed from the final model. Analyses were repeated in a subsample of participants with MCI at baseline in order to assess the effect of prediabetes in the prodromal stage of AD. The follow-up period for most AD markers was shorter in the MCI subsample given the smaller sample size at follow-up visits. Analyses were performed using SPSS 24 (SPSS Inc., Chicago, Illinois). Significance was defined as α = 0.05 (two-sided).

## Results

### Sample Characteristics

Prediabetes was more prevalent in men (*n* = 223, 45%) vs. women (*n* = 168, 40%), although the difference was not significant (χ^2^ = 2.5, *p* = 0.11). See Table 2 for sample characteristics and baseline AD markers by sex and prediabetic status. At baseline, women were younger with fewer years of education, a lower prevalence of past cardiovascular events, a lower mean baseline fasting blood glucose level, higher mean HV/IV ratio, and CMRglu. From a cognitive standpoint, women showed lower MMSE and AVLT scores compared to men (*p*s < 0.05) at baseline. Compared to normoglycemics, prediabetics had a significantly higher BMI and lower CMRglu at baseline and fewer years of follow-up (*p*s < 0.05). Among men only, the proportion of White participants was significantly lower in prediabetic vs. normoglycemic (*p* = 0.03). Among women only, the proportion of those with a history of CVD was significantly higher in prediabetics vs. normoglycemics (*p* = 0.02). Although BMI was higher in prediabetics vs. normoglycemics in both sexes, this difference was only significant in men (*p* = 0.04). In unadjusted analyses examining AD markers by sex and prediabetic status, baseline CMRglu was significantly lower in prediabetic vs. normoglycemic women (*p* = 0.005); however, baseline CMRglu did not differ between prediabetic and normoglycemic men (*p* = 0.21). Mean baseline executive function (TMT Part B) z-score was significantly lower in normoglycemic men vs. prediabetic men (*p* = 0.04). Baseline HV/IV, p-tau_181_/Aβ_1−42_ ratio, and episodic memory (AVLT) and language (BNT) z-scores did not differ by prediabetic status within men or women (*p*s ≥ 0.05). One-hundred ninety eight participants developed dementia during follow-up (73% MCI and 27% cognitively normal at baseline).

**Table 2:** Baseline sample characteristics and AD markers by sex and prediabetic status.

	**Women**			**Men**			**Sex** ***p*-value**	**Pre-diabetic Status** ***p*-value**
	**Normo-glycemic Mean (SD)**	**Prediabetic** **Mean (SD)**	***p-*value**	**Normo-glycemic Mean (SD)**	**Prediabetic** **Mean (SD)**	***p*-value**		
*N* (%)	251 (59.9%)	168 (40.1%)		269 (54.7%)	223 (45.3%)	0.11	–	–
Follow-up time, years	4.2 (2.4)	3.8 (2.4)	0.10	4.4 (2.9)	4.1 (2.6)	0.19	0.22	0.04
Age	72.2 (7.3)	72.2 (6.7)	0.93	73.9 (6.9)	73.9 (7.0)	0.95	<0.001	0.88
Education, years	15.8 (2.6)	15.7 (2.7)	0.74	16.7 (2.6)	16.5 (2.7)	0.28	<0.001	0.27
White, *N* (%)	229 (91.2%)	158 (94.0%)	0.16	258 (95.9%)	203 (91.0%)	0.03	0.40	0.55
APOE4 carrier, *N* (%)	102 (40.6%)	75 (44.6%)	0.42	117 (43.5%)	87 (39.0%)	0.31	0.85	0.85
MMSE score	28.5 (1.5)	28.3 (1.7)	0.28	28.2 (1.7)	28.1 (1.6)	0.69	0.02	0.31
MCI diagnosis, *N* (%)	104 (41.4%)	58 (34.5%)	0.16	111 (41.3%)	98 (43.9%)	0.55	0.22	0.62
Incident dementia cases, *N* (%)	55 (21.8%)	32 (19.2%)	0.51	67 (24.6%)	44 (19.7%)	0.19	0.50	0.16
Body mass index	26.5 (5.2)	27.4 (5.8)	0.08	26.8 (3.4)	27.5 (4.1)	0.04	0.47	0.006
History of CVD, *N* (%)	146 (58.2%)	117 (69.6%)	0.02	192 (71.3%)	152 (68.2%)	0.44	0.03	0.30
Pulse Pressure^*^ (mm Hg)	59.6 (18.3)	60.7 (14.7)	0.52	58.8 (13.0)	58.5 (14.8)	0.81	0.18	0.82
Baseline fasting blood glucose level (mg/dL)	87.8 (11.1)	107.8 (6.7)	<0.001	90.1 (8.5)	109.0 (6.5)	<0.001	0.002	<0.001
**AD biomarkers**								
HV/IV ratio	4.89 (0.75)	4.84 (0.78)	0.57	4.56 (0.75)	4.56 (0.72)	0.96	0.001	0.70
CMRglu	1.30 (0.13)	1.27 (0.13)	0.005	1.26 (0.14)	1.25 (0.12)	0.21	0.001	0.004
p-tau/Aβ ratio	0.03 (0.03)	0.03 (0.03)	0.78	0.03 (0.03)	0.03 (0.03)	0.43	0.75	0.76
**AD cognitive markers**								
AVLT Z score	−0.5 (1.2)	−0.3 (1.1)	0.34	−0.8 (1.0)	−0.9 (1.0)	0.48	<0.001	0.84
TMT Part B Z score	−0.4 (1.4)	−0.6 (1.5)	0.23	−0.6 (1.6)	−0.4 (1.2)	0.04	0.60	0.45
BNT Z score	−0.5 (1.4)	−0.7 (1.7)	0.18	−0.4 (1.5)	−0.5 (1.4)	0.37	0.13	0.12

*Table reflects sample characteristics of the largest sample examined (N = 911). “Sex p-value” and “Prediabetic Status p-value” columns reflect differences by sex across prediabetic status groups and differences by prediabetic status across sex, respectively. P-values within the sex columns reflect differences between prediabetes status within sex and are derived. All p-values are derived from univariate analyses of variance for continuous variables and Chi-square tests for categorical variables*.

* *Pulse pressure, systolic blood pressure (mm Hg) – diastolic blood pressure (mm Hg); MCI, Mild Cognitive Impairment; MMSE, Mini Mental State Exam; HV/IV, hippocampal/intracranial volume × 10^3^; CMRGlu, cerebral metabolic glucose rate; AVLT, Rey Auditory Verbal Learning Test; TMT, Trail Making Test; BNT, Boston Naming Test*.

**Figure 1:**
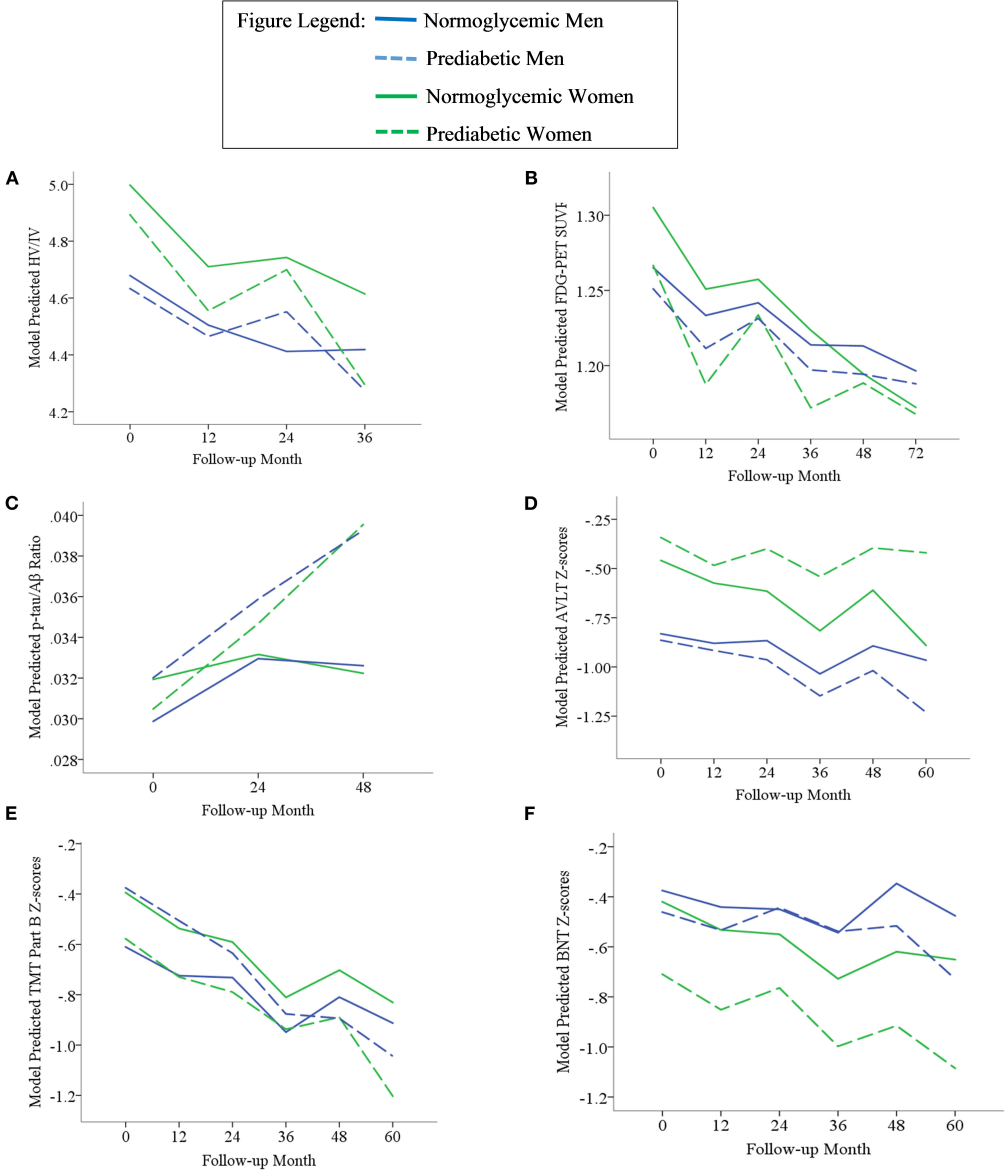
Trajectories of AD biomarkers **(A–C)** and cognitive test z-scores **(D–F)** by prediabetes status and sex in the overall sample. TMT Part B z-scores were multiplied by −1 so that higher scores indicated better performance. Z-scores are age- and education-adjusted based on a normative control group that remained cognitively normal throughout follow-up in ADNI. HV/IV, ratio of hippocampal volume to intracranial volume; PET, positron emission tomography; FDG, fludeoxyglucose; SUVR, standaradized uptake volume ratio; p-tau, phosphorylated tau; Aβ, amyloid beta; BNT, Bostin Naming Test; TMT, Trail Making Test; AVLT, Rey Auditory Verbal Learning Test.

**Figure 2:**
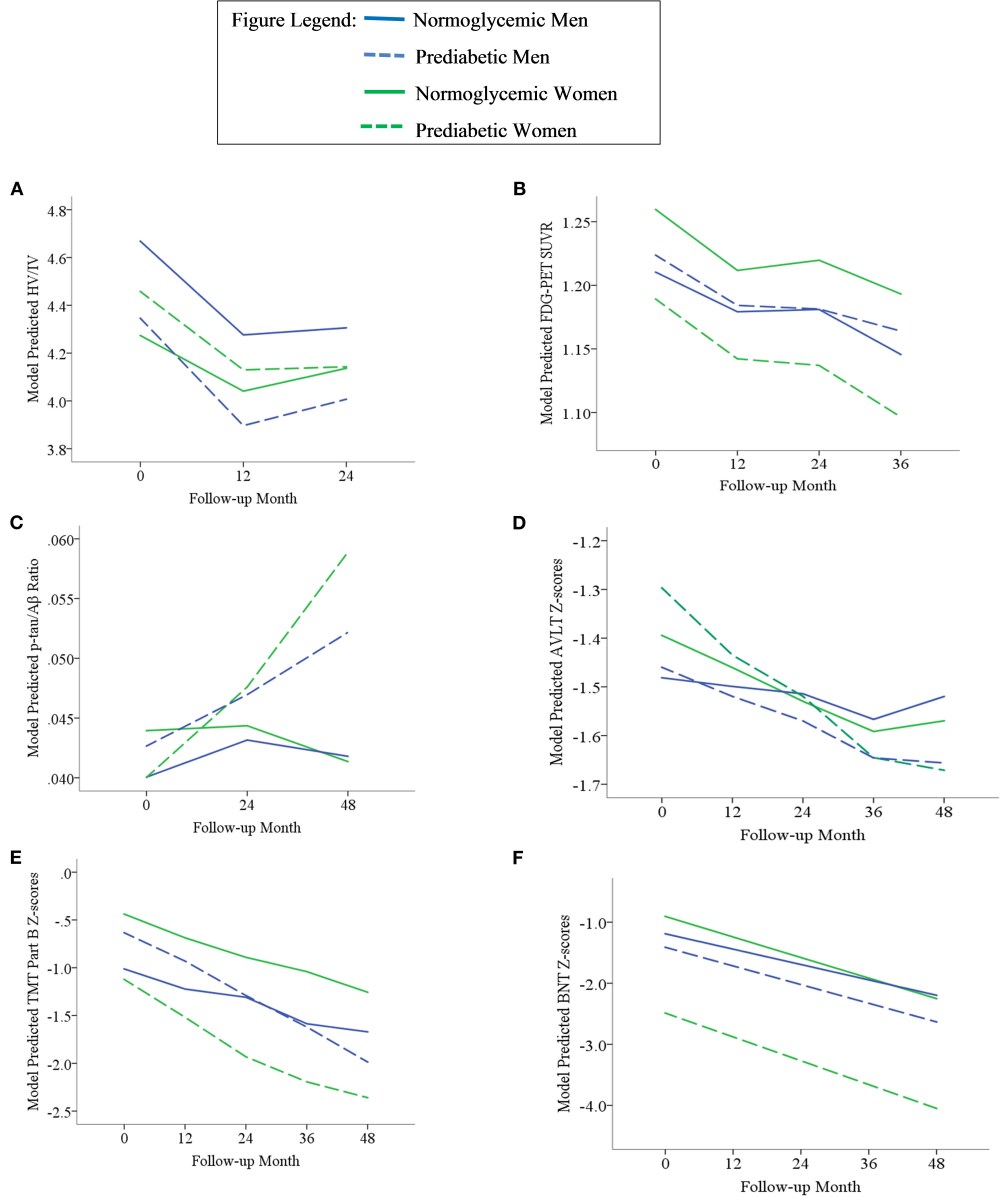
Trajectories of AD biomarkers **(A–C)** and cognitive test z-scores **(D–F)** by prediabetes status and sex in the MCI subsample. TMT Part B z-scores were multiplied by −1 so that higher scores indicated better performance. Z-scores are age- and education-adjusted based on a normative control group that remained cognitively normal throughout follow-up in ADNI. HV/IV, ratio of hippocampal volume to intracranial volume; PET, positron emission tomography; FDG, fludeoxyglucose; SUVR, standaradized uptake volume ratio; p-tau, phosphorylated tau; Aβ, amyloid beta; BNT, Bostin Naming Test; TMT, Trail Making Test; AVLT, Rey Auditory Verbal Learning Test.

Results of statistical tests modeling the interactive and separate effects of sex and prediabetes on the outcomes of (1) change in AD markers over time, (2) average AD marker level across time, and (3) risk of dementia and age at dementia onset are described below and separated by results in the overall sample vs. the MCI subsample. Parameter estimates from linear mixed effects regressions modeling AD markers over time are displayed in Table 3 (overall sample) and Table 4 (MCI subsample). The trajectories of AD markers by sex and prediabetic status are displayed in Figure 1 (overall sample) and Figure 2 (MCI subsample).

**Table 3:** Estimates of linear mixed effects models predicting change in AD markers by sex and prediabetes status in the overall sample.

	**Sex**			**Prediabetic Status**			**Time**			**Sex X prediabetes**			**Prediabetes X time**			**Sex X Time**			**Sex X Prediabetes X time**		
	**b**	**SE**	***p***	**b**	**SE**	***p***	**b**	**SE**	***p***	**b**	**SE**	***p***	**b**	**SE**	***p***	**b**	**SE**	***p***	**b**	**SE**	***p***
**AD biomarker**																					
HV/IV	−0.27	0.09	**0.003**	−0.14	0.09	0.13	−0.007	0.001	**<0.001**	0.11	0.18	0.53	0.001	0.001	0.51	0.001	0.001	0.33	0.001	0.002	0.71
CMRglu	−0.03	0.01	0.001	−0.04	0.01	**0.001**	−0.002	0.0002	**<0.001**	0.03	0.02	0.10	0.0004	0.0002	0.05	0.0004	0.0002	0.05	−0.3 × 10^−4^	0.0004	0.93
p-tau/ Aβ ratio	−0.003	0.003	0.20	−0.002	0.003	0.36	0.0001	0.3 × 10^−4^	**0.003**	0.005	0.003	0.17	0.0001	0.6 × 10^−4^	0.07	0.5 × 10^−4^	0.5 × 10^−4^	0.31	−0.0001	0.0001	0.26
**AD cognitive markers**																					
AVLT z-score	−0.36	0.05	**<0.001**	0.02	0.05	0.72	−0.004	0.0007	**<0.001**	−0.12	0.11	0.27	0.0002	0.001	0.90	0.002	0.001	0.18	−0.003	0.003	0.24
TMT Part B z-score	−0.18	0.12	0.14	−0.23	0.14	0.10	−0.009	0.002	**<0.001**	0.47	0.18	**0.01**	−0.003	0.002	0.27	0.001	0.002	0.63	−0.002	0.005	0.70
BNT z- score	0.20	0.08	**0.03**	−0.19	0.09	**0.03**	−0.006	0.001	**<0.001**	0.29	0.18	0.10	0.002	0.003	0.55	0.002	0.003	0.41	−0.001	0.005	0.84

*Bolded values indicate statistical significance. All analyses included the following covariates: age, education, race, APOE4 status, body mass index, pulse pressure and history of cardiovascular events. Covariates were removed from the final model if not a significant predictor of the outcome. SE, standard error; HV/IV, hippocampal/intracranial volume × 10^3^; CMRglu, cerebral metabolic glucose rate; AVLT, Rey Auditory Verbal Learning Test; TMT, Trail Making Test; BNT, Boston Naming Test*.

**Table 4:** Estimates of linear mixed effects models predicting change in AD markers by sex and prediabetes status in MCI subsample.

	**Sex**			**Prediabetic status**			**Time**			**Sex X prediabetes**			**Prediabetes X time**			**Sex X time**			**Sex X prediabetes X time**		
	**b**	**S.E**.	***p***	**b**	**S.E**.	***p***	**b**	**S.E**.	***p***	**b**	**S.E**.	***p***	**b**	**S.E**.	***p***	**b**	**S.E**.	***p***	**b**	**S.E**.	***p***
**AD biomarker**																					
HV/IV	−0.39	0.11	**<0.001**	−0.20	0.13	0.13	−0.01	0.0009	**<0.001**	0.19	0.17	0.26	−0.002	0.001	0.07	0.003	0.001	0.003	0.0002	0.002	0.94
CMRglu	−0.04	0.02	**0.04**	−0.06	0.02	**0.004**	−0.002	0.0003	**<0.001**	0.07	0.03	**0.02**	0.0002	0.0004	0.68	0.0001	0.0004	0.78	0.0006	0.0008	0.45
p-tau/ Aβ ratio	−0.002	0.003	0.54	0.0009	0.003	0.80	0.0001	0.00004	**<0.001**	0.01	0.007	0.08	0.0001	0.0001	0.23	−0.00001	0.0001	0.46	−0.0002	0.0002	0.26
**AD cognitive markers**																					
AVLT Z score	−0.07	0.08	0.41	0.006	0.08	0.94	−0.004	0.001	**<0.001**	−0.02	0.16	0.90	−0.003	0.002	0.14	0.003	0.002	0.11	0.003	0.004	0.40
TMT Part B Z score	−0.57	0.28	**0.04**	−0.69	0.34	**0.04**	−0.02	0.005	**<0.001**	1.07	0.46	**0.02**	−0.009	0.01	0.23	0.002	0.01	0.77	0.002	0.01	0.89
BNT Z score	−0.35	0.43	0.41	−1.58	0.53	**0.004**	−0.03	0.01	**0.006**	1.45	0.71	**0.04**	−0.004	0.01	0.75	0.007	0.01	0.60	0.002	0.03	0.94

*Bolded values indicate statistical significance. All analyses included the following covariates: age, education, race, APOE4 status, body mass index, pulse pressure, and history of cardiovascular events. Covariates were removed from the final model if not a significant predictor of the outcome. MCI, Mild Cognitive Impairment; HV/IV, hippocampal/intracranial volume × 10^3^. CMRglu, cerebral metabolic glucose rate; AVLT, Rey Auditory Verbal Learning Test; TMT, Trail Making Test; BNT, Boston Naming Test*.

### Sex x Prediabetes Interactive Effects in the Overall Sample and MCI Subgroup

#### Outcome: Change in AD Markers Over Time

In the overall sample and MCI subgroup, we found no Sex x Prediabetes interactive effects on change in AD markers (i.e., Sex x Prediabetes x Time interactions *p*s ≥ 0.05).

#### Outcome: Average AD Marker Across Time

In the overall sample, the Sex x Prediabetes interaction was significant only for executive function performance, whereby, on average, prediabetics performed significantly worse than normoglycemics, but only among women (B = −0.31, SE = 0.12, *p* = 0.01). Conversely, prediabetic men actually showed better executive function performance than normoglycemic men (B = 0.24, SE = 0.12, *p* = 0.04).

Within the MCI subgroup, there were significant Sex x Prediabetes interactions for the CMRGlu (*p* = 0.02), executive function (*p* = 0.02) and language outcomes (*p* = 0.04). The interactions revealed that, on average, prediabetic women exhibited lower CMRglu (B = −0.06, SE = 0.02, *p* = 0.005) and poorer executive function (B = −0.76, SE = 0.32, *p* = 0.02) and language performance (B = −1.69, SE = 0.54, *p* = 0.003) relative to normoglycemic women, whereas prediabetes did not relate to CMRglu (B = 0.01, SE = 0.02, *p* = 0.61), executive function (B = 0.41, SE = 0.31, *p* = 0.19) or language performance (B = −0.09, SE = 0.46, *p* = 0.84) in men.

#### Outcome: Incidence of Dementia and Age at Onset

Among participants with at-least one follow-up visit in the overall sample, (*N* = 850), the interactive effect of Sex x Prediabetes on risk of dementia was not significant (HR = 0.75, 95% CI = 0.36–1.54, *p* = 0.43). Among those who developed dementia, there was a significant Sex x Prediabetes interactive effect on age at dementia onset (B = −1.67, SE = 0.72, *p* = 0.02) that was unchanged when adjusting for baseline HV/IV and p-tau_181_/Aβ_1−42_ ratio but attenuated when adjusting for baseline CMRglu (B = −1.28, SE = 0.68, *p* = 0.06). The interaction was driven by a significant association between prediabetic status and age at dementia onset among women (B = −0.91, SE = 0.40, *p* = 0.03) but not men (B = 0.43, SE = 0.48, *p* = 0.37). Specifically, female prediabetics (M = 75.14, SD = 0.32) developed dementia at an earlier age than female normoglycemics (M = 76.05, SD = 0.25); however, this female-specific relationship of prediabetes to age at dementia onset was attenuated when adjusting for CMRglu (B = −0.75, SE = 0.41, *p* = 0.07).

In the MCI subgroup, the interactive effect of Sex x Prediabetes on risk of dementia was not significant (HR = 0.68, 95% CI = 0.29–1.60, *p* = 0.38). Among those who developed dementia, the Sex x Prediabetes interactive effect on age at dementia onset trended toward significance (B = −1.01, SE = 0.60, *p* = 0.09). This interaction became significant when adjusting for baseline HV/IV and p-tau_181_/Aβ_1−42_ ratio (B = −1.28, SE = 0.63, *p* = 0.04), but was attenuated with adjustment for CMRglu (B = −0.97, SE = 0.62, *p* = 0.012). The interaction was driven by a relationship between prediabetic status and age at dementia onset in women (B = −0.78, SE = 0.37, *p* = 0.04) but not in men (B = 0.54, SE = 0.48, *p* = 0.26), and this female-specific relationship was significant with and without adjustment for baseline AD biomarkers (HV/IV, p-tau_181_/Aβ_1−42_ ratio and CMRglu). Specifically, female prediabetics (M = 73.49, SD = 8.32) developed dementia at an earlier age than female normoglycemics (M = 74.31, SD = 6.72).

### Main Effects of Prediabetes and Sex in Overall Sample and MCI Subgroup

#### Outcome: Change in AD Markers Over Time

In the overall sample, a marginally significant Prediabetes x Time interaction (*p* = 0.05) indicated that CMRglu in normoglycemics declined faster over time from their higher baseline measure (B = −0.0015, SE = 0.0001, *p* < 0.001) compared to prediabetics (B = −0.0011, SE = 0.0001, *p* < 0.001). A marginally significant Sex x Time interaction (*p* = 0.05) indicated that CMRglu declined faster over time in women (B = −0.0016, SE = 0.0002, *p* < 0.001) compared to men (B = −0.0011, SE = 0.0001, *p* < 0.001). Although all AD markers showed significant progression over time (i.e., main effect of time, *p*'s <0.001), this change did not differ by prediabetic status or sex for any marker besides CMRglu.

Within the MCI subgroup, AD markers showed significant progression over time (i.e., main effect of time, *p*s < 0.01); however, this change did not differ by prediabetic status (i.e., non-significant Prediabetes X Time interactions, *p*s > 0.05). A significant Sex x Time interaction on HV/IV (*p* = 0.003) indicated that the initially higher HV/IV in MCI women showed faster decline over time (B = −0.012, SE = 0.001, *p* < 0.001) compared to MCI men (B = −0.008, SE = 0.001, *p* < 0.001). There was no effect of sex on change in other AD markers over time (*p*s > 0.05).

#### Outcome: Average AD Marker Across Time

In the overall sample, there were significant main effects of prediabetes on CMRglu (*p* = 0.001) and language performance (*p* = 0.03), whereby prediabetics demonstrated a lower average CMRglu and language z-score than normoglycemics across time points. There was a main effect of sex on HV/IV, episodic memory and language z-scores (*p*s < 0.05). Consistent with the broader literature (63–65), women demonstrated a higher average HV/IV and episodic memory z-score than men across time points. Conversely, women demonstrated a lower average language z-score compared to men across time points.

Within the MCI subgroup, there were main effects of prediabetic status on CMRglu, executive function and language outcomes in MCI, whereby, compared to normoglycemics, prediabetics demonstrated a lower average CMRglu, executive function z-score and language z-score across time points. There were also main effects of sex on HV/IV, CMRglu and executive function performance in the MCI subgoup, whereby women demonstrated a higher average HV/IV and CMRglu and a lower average executive function z-score than men across time points.

#### Outcome: Incidence of Dementia and Age at Onset

In the overall sample, there was no main effect of prediabetic status on risk of dementia (HR = 0.83, 95% CI = 0.61–1.14, *p* = 0.25). There was a main effect of sex on risk of dementia (HR = 0.72, 95% CI = 0.53–0.99, *p* = 0.04), whereby the risk was nearly 30% lower in men vs. women. Among those who developed dementia during follow-up, there was no main effect of prediabetic status (B = 0.44, SE = 0.43, *p* = 0.31) or sex (B = 1.51, SE = 0.95, *p* = 0.11) on age at dementia onset. Results were unchanged when adjusting for baseline AD biomarkers.

In the MCI subgroup, there were no main effects of prediabetic status (HR = 0.74, 95% CI = 0.48–1.15, *p* = 0.18) or sex (HR = 0.77, 95% CI = 0.51–1.17, *p* = 0.22) on risk of dementia. Similarly, among those who developed dementia during follow-up, there were no main effects of prediabetes (B = 0.50, SE = 0.42, *p* = 0.24) or sex (B = 1.44, SE = 0.94, *p* = 0.13) on age at dementia onset. Results were unchanged when adjusting for baseline AD biomarkers.

## Discussion

Among non-demented older adults, we found that prediabetes at baseline related to brain hypometabolism in both women and men; however, in line with our hypotheses, the adverse effects of prediabetes on cognitive outcomes were female-specific and limited to more frontal-mediated cognitive domains (i.e., executive function and language). Prediabetes was associated with poorer executive function performance in women only in the overall sample, whereas prediabetes curiously related to better executive function performance in men. When limiting analyses to those with MCI, the relationship between prediabetic status and hypometabolism became female-specific and the female-specific relationship between prediabetes and poorer executive function performance persisted, whereas the opposing relationship in men was no longer observed. Among those with MCI, we also observed a female-specific relationship between prediabetes and poorer language performance. Prediabetes did not relate to the p-tau_181_/Aβ_1−42_ ratio, HV/IV or memory performance in the overall sample or in the MCI subsample. In either the overall or MCI subsample, there was no effect of prediabetes on incident dementia rates regardless of sex; however, among incident dementia cases, prediabetes was associated with an earlier age of dementia onset in women but not in men.

The finding that brain hypometabolism was associated with prediabetes suggests that altered brain metabolism may be an early neural mechanism by which prediabetes/T2D aversely impacts cognition and AD risk. In fact, among incident dementia cases, we found that the significant Sex X Prediabetes interaction on age at dementia onset was attenuated when adjusting for CMRglu suggesting that brain hypometabolism may be a mechanism underlying what appears to be a hastening of the AD trajectory in female prediabetics vs. female normoglycemics. There is biological plausibility for this finding as animal studies have shown that impaired insulin modulation negatively affects brain glucose utilization (66, 67) likely through impeded delivery of glucose to the central nervous system [CNS; (68)]. Hypometabolism is characteristic of both prediabetes and AD and has been implicated in other AD mechanisms including tau protein hyperphosphorylation, vascular dysfunction, and inflammation (14, 15, 69). Thus, hypometabolism may represent a convergent pathway by which prediabetes predisposes one to AD brain changes. Our results in the overall sample showed significantly lower CMRglu in prediabetics throughout follow-up; however, a steeper rate of CMRglu decline over time in normoglycemics vs. prediabetics resulted in similar CMRglu at the final follow-up measure in prediabetics and normoglycemics. This pattern of results suggests that prediabetes may contribute to or compound the effects of AD and/or brain aging on physiological brain changes and, thus, accelerate these changes so that they occur earlier in the trajectory. Unlike results in the overall sample, the lower CMRglu in prediabetics vs. normoglycemics was consistent throughout follow-up in the MCI group. although the follow-up period examined was shorter in the MCI subsample. It is possible that we would have seen the normoglycemics decline faster to meet the already low rates in prediabetics with more follow-up time in the MCI group.

Our findings of an association between prediabetes and brain hypometabolism are consistent with preliminary findings from a previous, smaller study (*n* = 23) that reported an association between greater insulin resistance and hypometabolism in frontal, parietotemporal, and cingulate regions among prediabetics (14). Additionally, a previous ADNI study found an association between T2D and brain hypometabolism in MCI (13); however, prediabetes or sex differences were not examined. We saw no effect of prediabetes on HV/IV. Similarly, Schneider et al. (11) reported that smaller brain volume was associated with more severe diabetes (defined by higher HbA1c and longer disease duration) but not prediabetes or less-severe diabetes suggesting that the effects of insulin resistance on brain structure presumably do not manifest until a later disease stage (i.e., where structural changes are typically observed subsequent to altered brain function). We saw no significant effect of prediabetes on the p-tau_181_/Aβ_1−42_ ratio; however, we observed trends for a steeper increase in the ratio over time in the prediabetes vs. normoglycemic group in the overall sample and for a female-specific relationship between prediabetes and a higher ratio in the MCI subsample. These trends suggest that the effects of prediabetes on AD pathological markers and their sex differences may occur subsequent to effects on brain function and manifest with greater follow-up time. Consistently, a meta-analysis found that prediabetes was not associated with more advanced AD pathological markers in CSF (lower Aβ levels and higher p-tau and total tau levels) (18). However, in a subgroup analysis among studies with samples that were recruited through memory clinics, prediabetes was associated with lower Aβ levels and higher total tau, but not p-tau levels (18). Consistent with the idea that a relationship between prediabetes and AD pathological markers may manifest at a later AD stage, these clinic samples may represent more severe cognitive impairment than the MCI diagnosis in ADNI.

Regarding sex differences, we hypothesized that associations between prediabetic status and brain and cognitive outcomes would be stronger in women than in men. Results were partially consistent with hypotheses in that the associations of prediabetes with poorer executive function (overall sample and MCI subsample) and language performance (MCI subsample only) were not only stronger in women but were female-specific. In the overall sample, the relationship between prediabetes and brain hypometabolism across time was observed regardless of sex; however, prediabetes was associated with hypometabolism at baseline in women and not in men, and, in the MCI subsample, the longitudinal relationship became female-specific. One explanation for our sex disparities in findings may be that insulin resistance is more severe in women vs. men at the prediabetic stage and therefore shows more adverse effects. Arguing against this explanation, the mean fasting glucose level was actually lower in prediabetic women vs. prediabetic men in both the overall and MCI samples although not significantly (*p*s > 0.05). An alternative explanation is that women are more vulnerable than men to the adverse effects of a given level of insulin resistance. These more adverse effects of insulin resistance in women, possibly in combination with other early-stage AD brain changes, may surpass a threshold of brain insult that leads to greater cognitive deficits in prediabetics vs. normoglycemics among women. The reason for the sex disparity is unclear, although sex differences often suggest sex hormone mechanisms. Insulin concentrations have been reported to decline in men from the 5th to 8th decade of life but increase in women purportedly related to shifts in sex hormone status that occur with menopause (70). Another contributing factor that is related to prediabetes could be the higher percentage of body fat, particularly subcutaneous, and the adipose tissue derived leptin hormone in women vs. men (71, 72). Adiposity and its associated hormones are strongly associated with low-grade inflammation and this association is more robust in women vs. men (73–75) so that both normal and prediabetic women have shown higher levels of inflammatory markers than men (75).

Prediabetes related to poorer executive function and language performance only among women with MCI sample suggesting that prediabetes may only affect cognitive function among women on the AD trajectory. Considering that 83% of the MCI sample was biomarker positive for CSF Aβ and/or pTau levels, as determined based on established cutpoints (76), this suggests that the negative effect of prediabetes on cognition may only manifest when compounded by AD pathology. In line with this theory was our finding that prediabetic status did not affect rates of incident dementia in either sex but, among incident dementia cases, prediabetes was associated with an earlier age of dementia onset among women only. This might further suggest that prediabetes plays a role in the acceleration or severity of AD pathological cascades in women rather than the initiation of that cascade.

As hypothesized, prediabetes was associated with poorer executive function and language performance in women with MCI, but not with episodic memory performance, regardless of sex. This disparity could be because frontal-mediated cognitive impairments are commonly reported in relation to vascular mechanisms and vascular dementia (47–50). In support of our results is a meta-analysis of over 100,000 dementia cases that reported that T2D was a stronger risk factor specifically for vascular dementia in women compared to men (77). Cardiovascular risk factors in older adults are associated with microvascular changes in the cerebral white matter (78), which, regardless of brain location, are associated with diminished executive function (48). In addition, Type 2 diabetes and elevated pulse pressure have each been associated with declines in language abilities (2, 79). Disruptions of frontal-subcortical networks may lead to reduced abilities to retrieve semantic information, manifesting as word-finding difficulties. Similarly, a study from the National Alzheimer's Coordinating Center's Uniform Data examining longitudinal cognitive profiles in diabetic patients with MCI (*N* = 4,114) found that diabetes was associated with lower cognitive performance, primarily in non-memory domains (80). A more psychometric explanation for the association of prediabetes with language and executive functioning but not memory is also possible. The MCI group showed the poorest performance in the memory domain relative to other domains regardless of sex or prediabetes status, likely because ADNI targets the precursor stage to AD dementia—amnestic MCI—in their recruitment. The lower memory scores among the MCI participants may result in a restricted range that limits the ability to observe an effect of prediabetes.

There are study limitations. For example, the number of follow-up assessments differed by AD marker suggesting that our statistical power to detect significant effects varied across AD markers. We did not apply a statistical correction for our multiple comparisons; however, we felt this was not necessary given that we had apriori hypotheses concerning the specific outcomes that would relate to prediabetes and the specific direction of those relationships, with results mostly supporting hypotheses. In light of the observed effects in frontally-mediated cognitive domains, we would have ideally examined CMRglu specifically in more frontal regions; however, the number of ADNI participants with frontal lobe CMRglu in addition to our other variables of interest did not allow for statistically powered analyses. Lastly, ADNI is a convenience sample of mostly white and well-educated volunteers compared with the general US population, which limits the generalizability of results to the general population and particularly to other race/ethnic groups, which have differing rates of prediabetes and other cardiovascular comorbidities. Strengths of the current study include (1) a large sample that is well-characterized for AD neurocognitive deficits and biomarkers, (2) a longitudinal analyses of change in AD markers over time, (3) determining whether sex plays a moderating role in associations between prediabetes and AD-related outcomes, and (4) examination of prediabetes in relation to multiple cognitive domains and biomarkers of brain structure, function, and pathology.

In conclusion, although the adverse effects of T2D on brain and cognitive health are well documented, the effects of prediabetes on these outcomes are far less understood. Our results suggest that even prediabetes shows adverse effects on brain metabolism in older men and women. In light of previous findings relating T2D to reduced brain volume and AD pathology, our results suggest that functional changes may be seen before brain volume changes in the early trajectory of T2D. In women only, prediabetes was associated with poorer executive function overall and language performance specifically in the context of MCI. This suggests that women may be more susceptible than men to the negative effects of prediabetes on brain and cognition, and that these adverse effects may accelerate the progression of AD. Large-scale prospective studies are needed to further investigate sex disparities in the effect of prediabetes and T2D on other markers of brain health including white matter integrity and dementia progression rates. A large focus should be on modifiable risk factors such as prediabetes/T2D given that we still do not have any established treatments for progressive cognitive impairment. Practitioners should be advised that women with prediabetes may be at higher risk for cognitive decline and should target this population for careful assessment and intervention that could possibly delay the onset or progression of AD.

## Data Availability Statement

The original contributions presented in the study are included in the article/supplementary material, further inquiries can be directed to the corresponding author/s.

## Ethics Statement

The studies involving human participants were conducted by the Alzheimer's Disease Neuroimaging Initiative (ADNI), and were approved by the Institutional Review Boards of all participating sites. Participants provided their written informed consent to participate in the study.

## Author Contributions

ES and LD-W conceived and designed the research project. ES, KT, and AW aggregated the data. ES conducted statistical analyses, wrote the paper, and has primary responsibility for final content. All authors consulted on study design, statistical analyses, manuscript editing, read, and approved the final manuscript.

## Conflict of Interest

MB is a consulting editor for the Journal of the International Neuropsychological Society, and serves as a paid consultant for Novartis, Eisai, and Roche pharmaceutical companies. The remaining authors declare that the research was conducted in the absence of any commercial or financial relationships that could be construed as a potential conflict of interest. The handling editor is currently organizing a Research Topic with one of the authors ES.
